# An artificial neural network model to diagnose non-obstructive azoospermia based on RNA-binding protein-related genes

**DOI:** 10.18632/aging.204674

**Published:** 2023-04-24

**Authors:** Fan Peng, Bahaerguli Muhuitijiang, Jiawei Zhou, Haoyu Liang, Yu Zhang, Ranran Zhou

**Affiliations:** 1 Department of Urology, Baoan Central Hospital of Shen Zhen, Shenzhen 518102, China; 2 Department of Urology, Nanfang Hospital, Southern Medical University, Guangzhou 510000, China; 3 The First School of Clinical Medicine, Southern Medical University, Guangzhou 510000, China; 4 Department of Urology, The Third Affiliated Hospital, Southern Medical University, Guangzhou 510000, China

**Keywords:** machine learning, artificial neural network, diagnosis, non-obstructive azoospermia, RNA-binding protein

## Abstract

Non-obstructive azoospermia (NOA) is a severe form of male infertility, but its pathological mechanisms and diagnostic biomarkers remain obscure. Since the dysregulation of RNA-binding proteins (RBPs) had nonnegligible effects on spermatogenesis, we aimed to investigate the functions and diagnosis values of RBPs in NOA. 58 testicular samples (control = 11, NOA = 47) from Gene Expression Omnibus (GEO) were set as the training cohort. Three public datasets, containing GSE45885 (control = 4, NOA = 27), GSE45887 (control = 4, NOA = 16), and GSE145467 (control = 10, NOA = 10), and 44 clinical samples from the local hospital (control = 27, NOA = 17) were used for validation. Through a series of bioinformatical analyses and machine learning algorithms, including genomic difference detection, protein-protein interaction network analysis, LASSO, SVM-RFE, and Boruta, DDX20 and NCBP2 were determined as significant predictors of NOA. Single-cell RNA sequencing of 432 testicular cell samples from NOA patients indicated that DDX20 and NCBP2 were associated with spermatogenesis (false discovery rate < 0.05). Based on the transcriptome expressions of DDX20 and NCBP2, we constructed multiple diagnosis models using logistic regression, random forest, and artificial neural network (ANN). The ANN model exhibited the most reliable predictive performance in the training cohort (AUC = 0.840), GSE45885 (AUC = 0.731), GSE45887 (AUC = 0.781), GSE145467 (AUC = 0.850), and local cohort (AUC = 0.623). Totally, an ANN diagnosis model based on RBP DDX20 and NCBP2 was developed and externally validated in NOA, functioning as a promising tool in clinical practice.

## INTRODUCTION

As the most grievous situation of male infertility, azoospermia refers to the complete absence of spermatozoa in the ejaculate. The prevalence of azoospermia is fairly high, accounting for 1% of males and over 10% of infertile males [1]. Around 30% of azoospermia patients exhibit obstructive azoospermia (OA) due to the physical blockage of the sperm outflow tract, and these cases usually show normal spermatogenesis. Non-obstructive azoospermia (NOA), which accounts for 70% of the azoospermia cases, is characterized by spermatogenetic failure and testicular dysfunction [2]. NOA is mainly caused by various genetic diseases, adverse drug effects, and malignant tumors [2, 3]. Therefore, the surgical removal of the blockage and the testicular puncture are valid in OA, while the current treatment strategies for NOA often fail [4]. Seeking the underlying biological mechanisms of NOA has attracted increasing attention in the past decades, and some critical genes were uncovered, such as VASA [5], CHD5 [6], and MEIOB [7], providing possible therapeutic targets. Nevertheless, our understandings of the genetic alternation of NOA remain limited. A prominent manifestation is that the examination of any NOA-specific gene has not been recommended in current clinical guidelines [8–10].

RNA-binding proteins (RBPs) are a group of proteins capable of regulating a plethora of cellular post-transcriptional processes, the perturbation of which leads to impaired spermatogenesis. Previous studies have demonstrated that RBPs tremendously influence the mammalian reproductive system. For instance, RBP Rbm46 knockout mice had reduced testes size and spermatogenetic defect and thus were infertile [11]; Boule was able to bind to a tremendous amount of spermatogenesis-related mRNAs and was involved in the spermatogenetic process in mice testes through forming amyloid-like aggregation both *in vivo* and *in vitro* [12]; The loss of RBP Tulp2 led to infertility in male mice by reducing the quantity and quality of sperms [13]. Given that a large number of proteins need to be properly expressed during spermatogenesis, it is inevitable that RBPs exert nonnegligible functions in the spermatogenetic process and, naturally, in the initiation and progression of NOA. However, the number of studies focusing on the relationship between RBPs and NOA is currently inadequate.

The present study collected the RBPs from previous reports and compared their expression levels between control and NOA testicular samples. Multiple bioinformatical and machine learning algorithms, including protein-protein interaction (PPI) network analysis, least absolute shrinkage and selection operator (LASSO), support vector machine-recursive feature elimination (SVM-RFE), and Boruta, were adopted for feature selection. Logistic regression (LR), random forest (RF), and artificial neural network (ANN) were harnessed to construct the diagnosis models. The GSE9210 dataset obtained from Gene Expression Omnibus (GEO) was set as the training cohort, while GSE45885, GSE45887, and GSE145467 from GEO were used for external validation. Importantly, we collected the seminal plasma and testicular tissue of 27 control and 17 NOA samples from the local hospital to re-confirm the reliability of the models. Single-cell RNA sequencing (scRNA-seq) data of 432 testicular cell samples from NOA patients were used to investigate the association of the unearthed genes and spermatogenesis.

## MATERIALS AND METHODS

### Data retrieval and processing

A sum of 1542 RBPs was gleaned from the report of Gerstberger et al. [14], as listed in Supplementary Table 1. The transcriptome sequencing data of the testicular tissue from 11 control and 47 NOA patients in the GSE9210 dataset [15] was directly downloaded from GEO (https://www.ncbi.nlm.nih.gov/geo/) as the training dataset. At the same time, GSE45885 [16] (control = 4, NOA = 27), GSE45887 [17] (control = 4, NOA = 16), and GSE145467 (control = 10, NOA = 10) were obtained from GEO as the external validation datasets. The control cases were defined as the subjects with normal spermatogenesis, including healthy donors and OA patients. The chip probe IDs were converted into gene symbols using R software (version 4.1.0) following the annotation files. The average expression value would be adopted if multiple probe IDs corresponded to the same gene symbol. The RNA expression values in these cohorts were all normalized with log2 (*x* + 1) transformation, and the sva package in R software was used to reduce the batch effects of these experiments as possible.

The scRNA-seq of 432 testicular cell samples from NOA patients was obtained from GEO (GSE157421) [18] to investigate the association of the screened genes with spermatogenesis. The processing of analyses of the scRNA-seq was described in the previous study in detail [19]. More information on these public datasets mentioned above is shown in Table 1.

**Table 1:** The detailed information of the public datasets from GEO.

**ID**	**Platform**	**Experimental type**	**Tissue**	**Control**	**NOA**	**Region**
GSE9210	GPL887	Microarray	Testes	11	47	Japan
GSE45885	GPL6244	Microarray	Testes	4	27	Norway
GSE45887	GPL6244	Microarray	Testes	4	16	Norway
GSE145467	GPL4133	Microarray	Testes	10	10	Unknown
GSE157421	GPL20301	Single-cell RNA sequencing	Testes	–	432	China

Abbreviations: GEO: gene expression omnibus; NOA: non-obstructive azoospermia.

### Clinical sample collection

A total of 44 participants, including 27 cases with normal spermatogenesis (OA) and 17 NOA subjects, were enrolled in this project between January 2021 and May 2022 at the Bao’an Central Hospital of Shenzhen (China). The study protocol was reviewed and approved by the Ethics Committee of Bao’an Central Hospital of Shenzhen, and all the patients signed the informed consent. The paraffin-embedded testicular biopsy specimens of these patients were provided by the Department of Pathology in Bao’an Central Hospital of Shenzhen. The semen samples were collected by masturbation following 3–5 days of sexual abstinence. The seminal supernatant plasma was obtained after centrifuging semen at 3,000 g for 20 min and then immediately stored at −80°C for RNA extraction. Other critical clinicopathological parameters, inclusive of age, Johnsen’s Score, follicle-stimulating hormone (FSH) levels, luteinizing hormone (LH) levels, and testosterone (T) levels, were retrospectively documented as well.

### Real-time quantitative PCR experiments

We conducted real-time quantitative PCR (RT-qPCR) experiments to quantify the mRNA expression levels of the screened genes in the seminal plasma of the local cohort. The total RNA of the seminal plasma samples was isolated using TRIzol Reagent (Invitrogen, USA) following the manufacturer’s protocols. The PrimeScript RT Reagent Kit (Takara, China) was used to perform the reverse transcription. The PCR experiments were then carried out based on ABI 7600 system (Applied Biosystems, USA) with the SYBR Premix ExTaq kit (Takara, China). GAPDH was chosen as the internal reference gene, and all the detected values were normalized with the 2−ΔΔCt method. The primer sequence of GAPDH, DDX20, and NCBP2 was designed and synthesized by the TSINGKE Company (Guangzhou, China), which is shown in Table 2.

**Table 2:** The primer sequence used in this study.

**Gene**	**Sequence (5′–3′)**
NCBP2	F: AAAACGCCATGCGGTACATAA
	R: GCCTGCCCTCCTTAAAGCC
DDX20	F: GCTGCGGGCTCGATTTAATTG
	R: GTCCAAAGCTATGGTGGAGAAC
GAPDH	F: GGAGCGAGATCCCTCCAAAAT
	R: GGCTGTTGTCATACTTCTCATGG

### Immunohistochemical staining

The immunohistochemical (IHC) staining of the paraffin-embedded testicular samples of the local cohort was implemented to investigate the protein expression levels and distribution of DDX20. The testicular slides were de-paraffinized in xylene and then added to the ethanol following the below concentration: 100% ethanol (4 min), 90% ethanol (4 min), 80% ethanol (4 min), and 70% ethanol (4 min). Subsequently, the slides were blocked in phosphate-buffered saline (PBS) supplemented with 5% bovine serum albumin (BSA) for 1 hour at room temperature and incubated with the primary antibody (rabbit anti-DDX20, 1:100, Proteintech, China) at 4°C overnight. After washing the slides 3 times with PBS, we incubated the slices with anti-rabbit secondary antibodies (Proteintech, China). Nikon Eclipse 90i system (Nikon, Japan) was used to get the images, and the Image-Pro Plus (version 6.0, Media Cybernetics, USA) software was used to measure the integral optical density (IOD), which represented the protein expression levels. For each sample, 3 slices were randomly chosen to conduct the IHC staining, and 5 different microscopic fields of a slide were randomly selected to evaluate the levels of DDX20.

### Gene expression difference analysis and functional annotation

The mRNA expression divergence of the 1542 RBPs between the control and NOA testicular samples was detected with the limma package in R. The filtering criteria were set as follows: |logFC| > 1 and false discovery rate (FDR) < 0.05. The biological gene function enrichment was performed using the Metascape online tool (https://metascape.org/) to identify the associated Gene Ontology (GO) terms and Kyoto Encyclopedia of Genes and Genomes (KEGG) pathways with *P* < 0.05 filtering threshold.

### PPI network construction and analysis

We uploaded the differentially-expressed RBPs to the STRING database (https://string-db.org/) to construct the PPI network to investigate the interaction of these genes. The confidence level was set to 0.4, and the genes without association with other nodes were excluded. The Cytoscape software (version 3.8.0) was utilized to visualize the PPI network. The cytoHubba plug-in in the Cytoscape software was used to measure the importance of the genes in the network. We chose the Top 20 genes showing the highest degree for further analyses.

### Feature selection via machine learning algorithms

We employed multiple feature selection algorithms to investigate the significant diagnosis biomarkers in NOA, including LASSO, SVM-RFE, and Boruta. LASSO Binomial regression with nested cross-validation to select the optimal predictor was built using the glmnet R package. SVM-RFE algorithm, which was based on the backward feature elimination that recursively removes the least ranking feature, was conducted by the caret package. The Boruta algorithm, which was built around the random forest, was also used to remove the irrelevant and redundant features through the Boruta package in R, where the variables labelled with “Confirmed” were identified. Following these results, we included the RBPs con-determined by LASSO, SVM-RFE, and Boruta in the diagnosis model development.

### Construction and validation of diagnosis models using LR, RF, and ANN

We implemented 3 commonly used machine learning-based methods to construct the diagnosis models, including LR, RF, and ANN, to improve the predictive power and robustness. The LR model was developed based on the “glm” function with default settings in R software. The RF model was established using the randomForest package with the following parameters: ntree = 500, mtry = 3, importance = T, and proximity = T. The ANN model was constructed according to the neuralnet R package, which contained one input layer, one hidden layer, and one output layer. In the hidden layer, we applied 5 nodes, and rectified linear unit was utilized as an activation function. Two nodes (control and NOA) were set in the output layer, where a softmax function was employed. According to the ANN model, the classification score of each subject was calculated.

To ensure the comparability of the LR, RF, and ANN models, we regarded that the sample would be classified as a control case if its probability predicted by the LR and ANN models was less than 0.5; otherwise, it would be considered an NOA sample. The receiver operating characteristic (ROC) analyses were performed to measure the predictive performance of the models in different cohorts through the pROC package. The confusion matrices, and other statistical indexes, such as accuracy, precision, recall, F-measure, sensitivity, specificity, positive predictive value, and negative predictive value, were also applied in this study.

### The functionally-related genes

The Top 20 genes interacting with the screened genes were obtained from the GeneMANIA database (http://genemania.org/) with default settings. The interaction types included physical interactions, co-expression, prediction, co-localization, genetic interactions, pathways, and shared protein domains.

### Gene set enrichment analysis

Here, we adopted the single-gene gene set enrichment analysis (GSEA) strategy to investigate the association between the screened genes and spermatogenesis. According to the median expression value of the particular gene, 432 testicular cell samples were divided into high- and low-gene expression groups, followed by the GSEA analysis. The GSEA was conducted via the GSEA software (version 4.1.0) with default settings, and the reference gene sets (Hallmark version 7.2) were downloaded from the Molecular Signature Database (https://www.gsea-msigdb.org/gsea/msigdb/). The term with Nominal *P* < 0.05 and FDR < 0.05 was considered to be statistically significant.

### Statistical analyses

The statistical analyses of the whole study were based on the R software (version 4.1.0) and GraphPad Prism 8 (version 8.4.3). The data in this study are presented as n (%) or mean ± standard deviation (SD). The two-tailed Student’s *t*-test was used to compare the difference in the RT-qPCR experiments, and The Welch-corrected *t*-test was used for IODs. Unless otherwise specified, *P* < 0.05 was significant. ^*^P < 0.05; ^**^P < 0.01; ^***^P < 0.001.

## RESULTS

### 51 RBPs were differentially expressed between control and NOA samples

**Figure 1:**
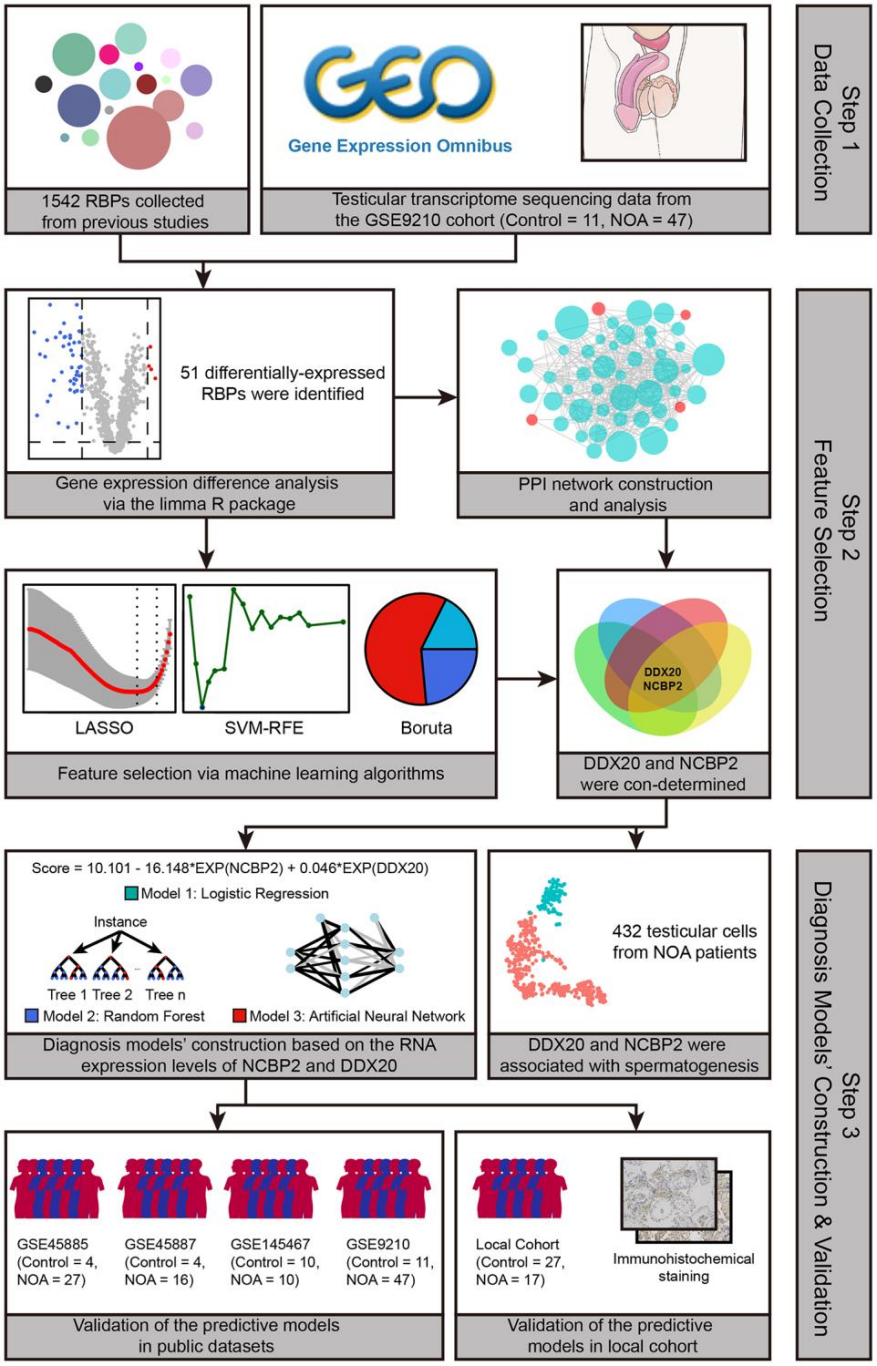
The workflow of the present study.

**Figure 2:**
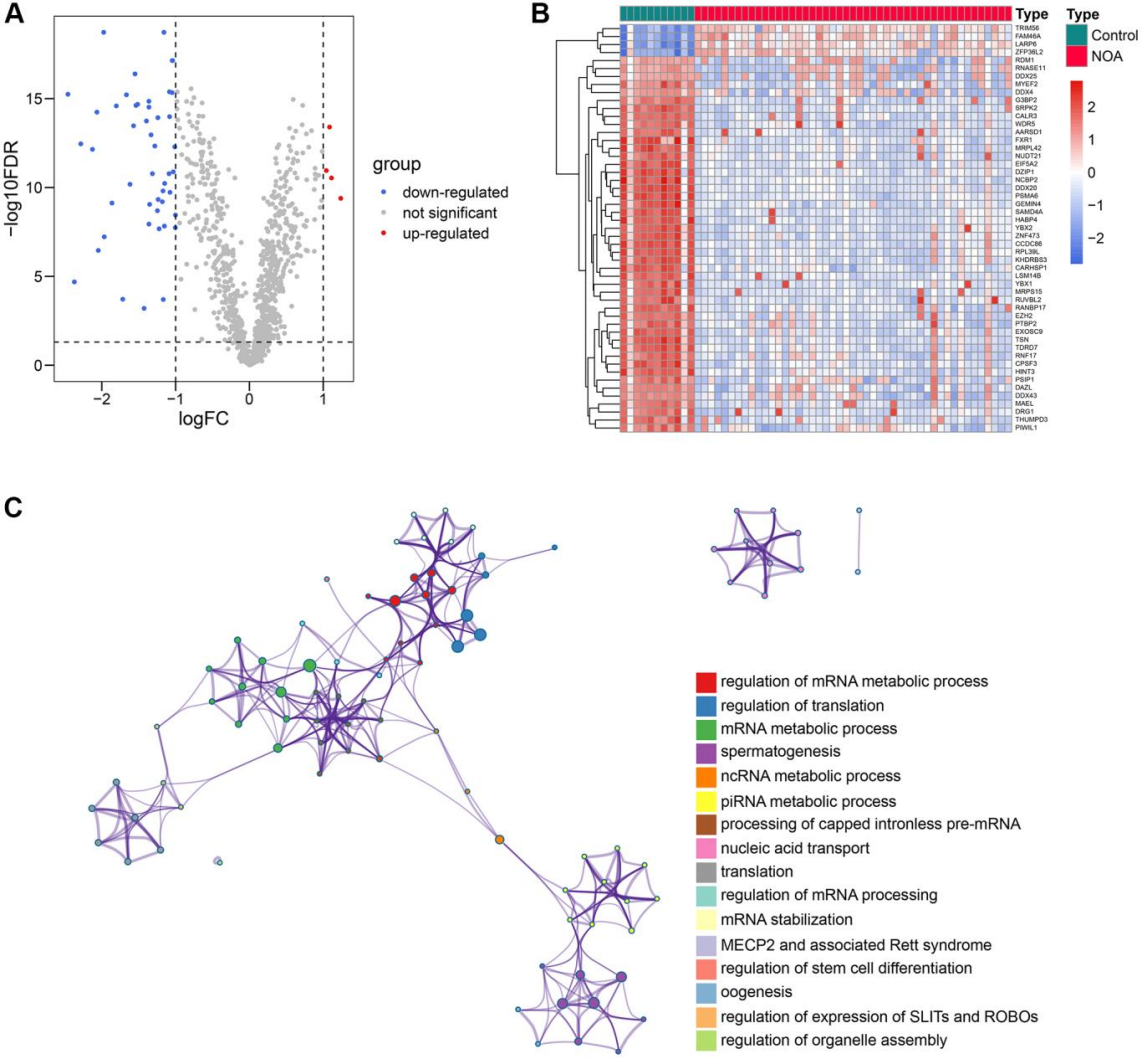
**51 differentially-expressed RBPs and their functional enrichment.** (**A**, **B**) The volcano plot (**A**) and the heat map (**B**) indicated that 51 of 1542 RBPs were differentially expressed between the control and NOA testicular samples. (**C**) The functional annotation of the 51 RBPs. Abbreviations: RBP: RNA-binding protein; NOA: non-obstructive azoospermia.

The schematic workflow chart, which graphically describes the methodology of this study, is presented in Figure 1. First, we compared the mRNA expression levels of 1542 RBPs collected from previous studies in the testicular samples of control and NOA cases in the GSE9210 cohort. The results indicated that 51 of 1542 RBPs were differentially expressed (Supplementary Table 2), as shown in the volcano plot (Figure 2A) and heat map (Figure 2B). The functional annotation displayed that the 51 differentially-expressed genes (DEGs) were mainly involved in translation regulation, RNA metabolism, RNA stability regulation, and spermatogenetic process, implying the tremendous effect of RBPs on the pathogenesis of NOA (Figure 2C).

### PPI network construction

**Figure 3:**
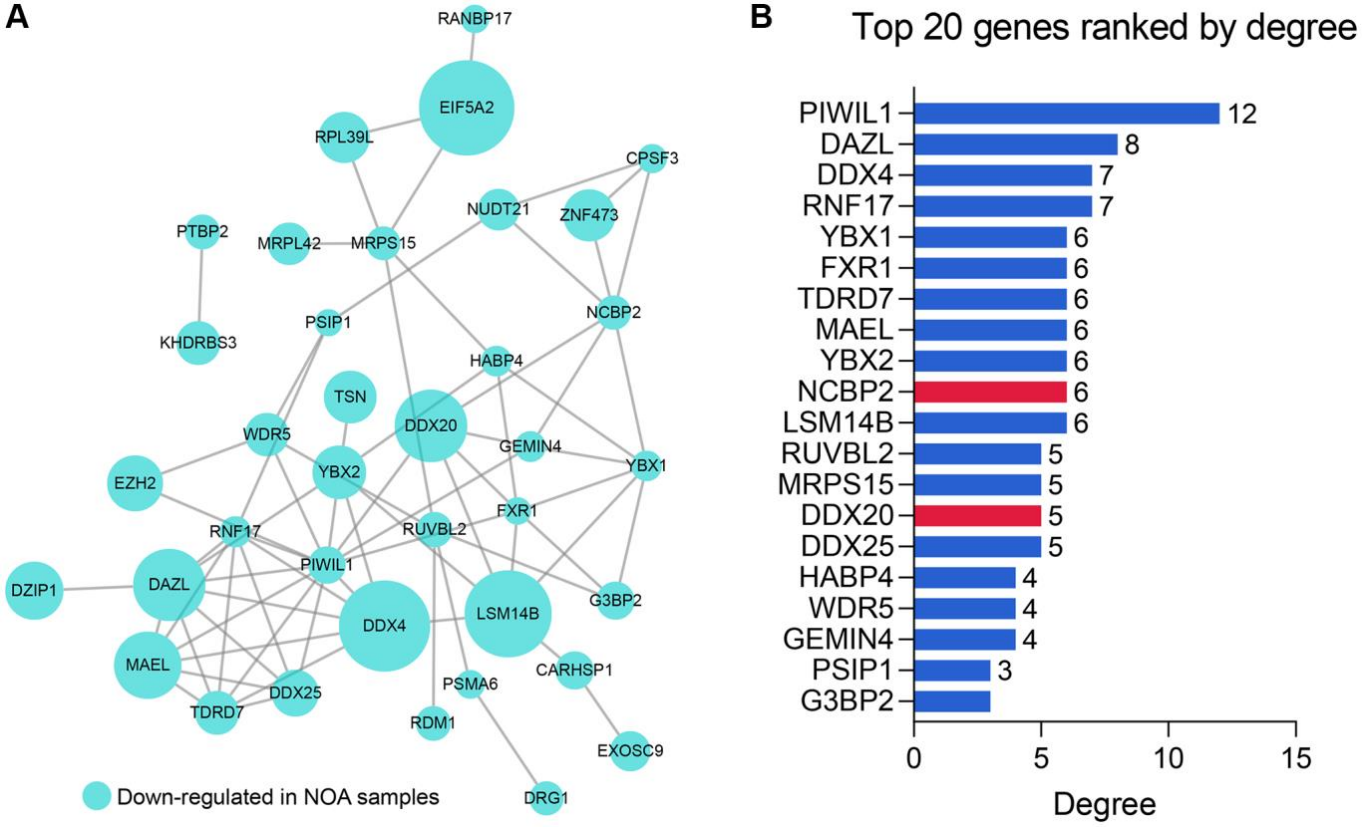
**The PPI network analysis of the 51 RBPs.** (**A**) The PPI network of the 51 RBPs, where the genes unconnected with other genes were excluded. (**B**) The Top 20 genes with the highest degree in the PPI network. Abbreviation: PPI: protein-protein interaction network.

We constructed the PPI network to further explore the internal contact and interactions among the 51 DEGs at the protein level. Figure 3A illustrates the established PPI network, where the size of the nodes represented the absolute value of their corresponding logFC in the GSE9210 cohort. The importance and influence of the genes in the network were quantified as degrees, and the Top 20 genes with the highest degree were identified and selected for the next step of research (Figure 3B, Supplementary Table 3).

### DDX20 and NCBP2 were identified via feature selection algorithms and PPI network analysis

**Figure 4:**
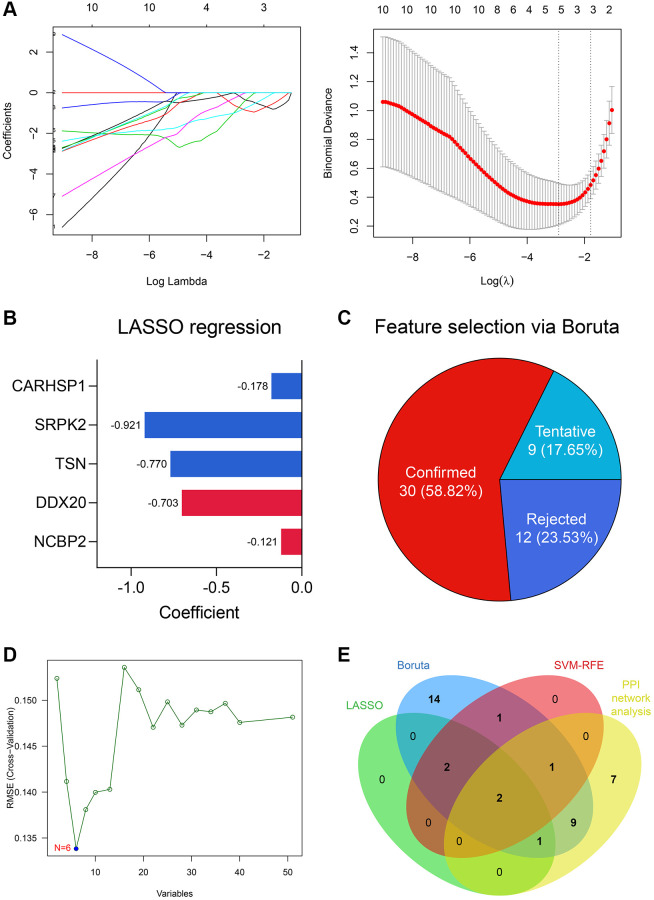
**DDX20 and NCBP2 were con-determined via feature selection methods and PPI network analysis.** (**A**) 5 genes, including NCBP2, DDX20, TSN, SRPK2, and CARHSP1, were identified as significant features to NOA via LASSO regression. (**B**) The coefficients of the 5 selected genes in the LASSO regression model. (**C**) 30 genes were determined as important features via the Boruta algorithm. (**D**) 6 genes, including NCBP2, DDX20, CCDC86, TSN, CARHSP1, and TDRD7, were selected by the SVM-RFE. (**E**) DDX20 and NCBP2 were con-determined by the machine learning algorithms and PPI network analysis.

5 genes were identified as important features of NOA through LASSO regression (Figure 4A), including NCBP2, DDX20, TSN, SRPK2, and CARHSP1. The coefficients of these genes in the LASSO regression model were −0.121, −0.703, −0.770, −0.921, and −0.178, respectively (Figure 4B). Simultaneously, 30 of 51 DEGs were selected via the Boruta algorithm (Figure 4C, Supplementary Table 4), and 6 genes, including NCBP2, DDX20, CCDC86, TSN, CARHSP1, and TDRD7, were screened by SVM-RFE (Figure 4D). Ultimately, NCBP2 and DDX20 were identified by integrating the Top 20 genes with the highest degree in the PPI network and these feature selection results (Figure 4E) and then included in the diagnosis model construction.

### External validation of DDX20 and NCBP2

**Figure 5:**
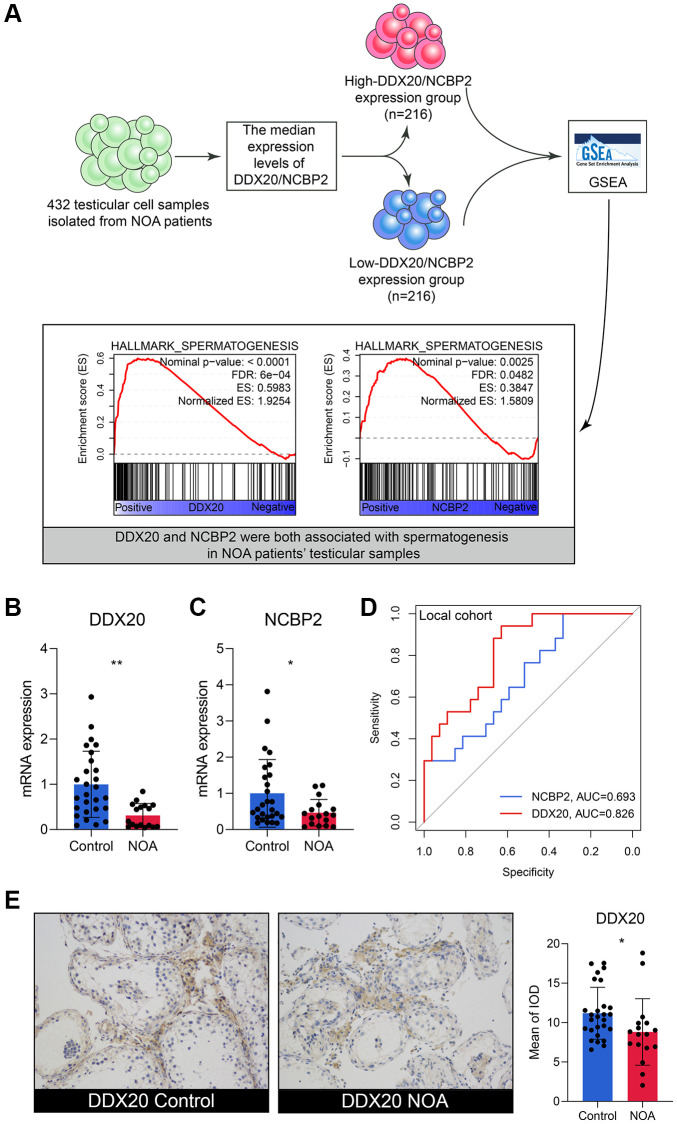
**The external validation of DDX20 and NCBP2.** (**A**) The single-cell RNA-sequencing analysis of 432 testicular cell samples isolated from NOA patients displayed that DDX20 and NCBP2 were both positively associated with spermatogenesis. (**B**, **C**) DDX20 (**B**) and NCBP2 (**C**) were down-regulated in the seminal plasma samples of NOA patients from the local hospital. (**D**) The ROC curve exhibited the diagnosis ability of seminal plasma DDX20 and NCBP2 to NOA in the local cohort. (**E**) The protein expression levels of DDX20 were obviously down-regulated in the testicular samples from NOA patients in the local cohort, which were detected by the immunohistochemical staining. Abbreviation: ROC: receiver operating characteristic.

The scRNA-seq data of 432 testicular cell samples indicated that DDX20 (Nominal *P* < 0.001, FDR < 0.001) and NCBP2 (Nominal *P* < 0.01, FDR < 0.05) were both positively associated with the spermatogenetic process (Figure 5A), re-confirming that DDX20 and NCBP2 were significant biomarkers to NOA. Next, we gleaned the seminal plasma and testicular biopsy of 27 control and 17 NOA patients from the local hospital for validation. Compared with the control samples, the NOA samples exhibited lower mRNA levels of DDX20 (*P* < 0.01, Figure 5B) and NCBP2 (*P* < 0.05, Figure 5C) in seminal plasma, suggesting that the levels of DDX20 and NCBP2 in seminal plasma were also promising diagnostic biomarkers for NOA. ROC analysis showed that DDX20 in seminal plasma was a powerful classifier for NOA (area under the curve [AUC] = 0.826, 95% confidence interval [CI] = 0.706–0.946, Figure 5D), while the predictive performance of NCBP2 was relatively low (AUC = 0.693, 95% CI = 0.534–0.852, Figure 5D), which might be caused by the heterogeneity across different cohorts. Hence, we then investigated the protein levels of DDX20 in the local cohort using IHC staining, and the results supported the conclusion drawn before that DDX20 was significantly down-regulated in NOA testicular samples (*P* < 0.05, Figure 5E).

### The performance of LR, RF, and ANN diagnosis models

The present study utilized multiple datasets, including GSE9210, GSE45885, GSE45887, and GSE145467, and local clinical samples to verify the predictive ability of the established model. The detailed clinicopathological parameters of the training and external validation cohorts are displayed in Table 3. It should be stated that we used the mRNA expression values in the seminal plasma, other than in the testicular samples, to validate the models in the local cohort because the fresh testicular samples were unavailable given the policy formulated by the ethics committee of our hospital. Since we have detected the expressions of DDX20 and NCBP2 in the seminal plasma and found that both genes were down-regulated in NOA samples, which corresponded to the results in the training dataset, we thought that the validation in the seminal plasma samples from the local cohort was still acceptable.

**Table 3:** The clinicopathological features of the cohorts enrolled in this study.

**Characteristics**	**GSE9210**		**GSE45885**		**GSE45887**		**GSE145467**		**Local cohort**	
	**Control (*n* = 11)**	**NOA (*n* = 47)**	**Control (*n* = 4)**	**NOA (*n* = 27)**	**Control (*n* = 4)**	**NOA (*n* = 16)**	**Control (*n* = 10)**	**NOA (*n* = 10)**	**Control (*n* = 27)**	**NOA (*n* = 17)**
Age (years)	33.3 ± 8.5	35.0 ± 5.7	–	32.1 ± 4.05	–	31.3 ± 1.8	–	–	31.8 ± 9.2	33.4 ± 7.4
Johnsen’s score	7.9 ± 1.2	2.4 ± 1.3	–	4.9 ± 2.5	–	–	–	–	7.3 ± 1.7	2.7 ± 2.5
FSH (mIU/ml)	10.1 ± 9.3	29.2 ± 9.1	–	–	–	–	–	–	11.1 ± 7.6	21.6 ± 8.8
LH (mIU/ml)	4.5 ± 2.3	8.8 ± 4.8	–	–	–	–	–	–	4.3 ± 0.9	8.1 ± 3.5
T (ng/ml)	4.8 ± 1.7	3.5 ± 1.6	–	–	–	–	–	–	5.1 ± 0.7	3.7 ± 1.2

Abbreviations: FSH: follicle-stimulating hormone; LH: luteinizing hormone; T: testosterone; NOA: non-obstructive azoospermia.

**Figure 6:**
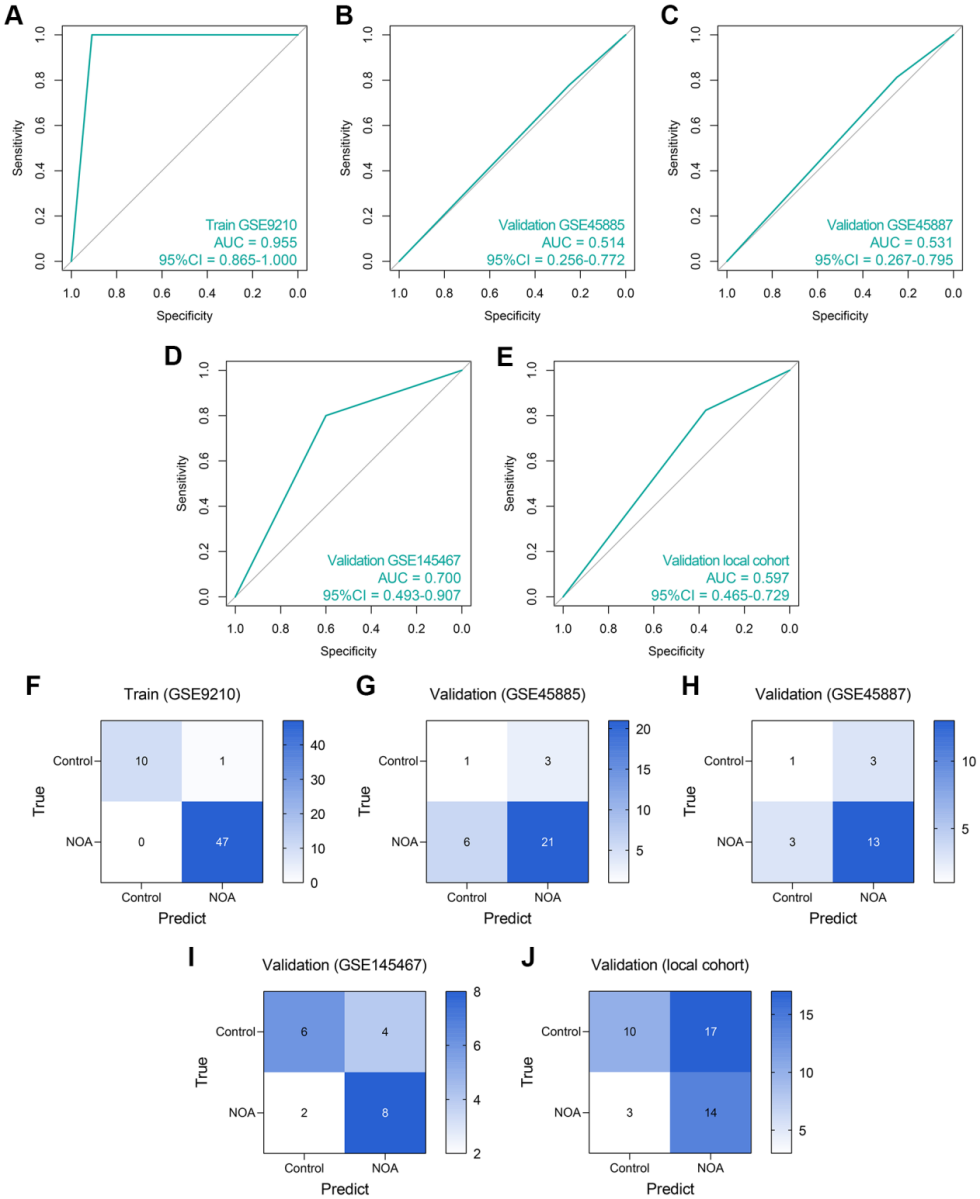
**The predictive performance of an LR diagnosis model in each cohort.** (**A**–**E**) The ROC analyses of the LR model in the training cohort (**A**), GSE45885 cohort (**B**), GSE45887 cohort (**C**), GSE145467 cohort (**D**), and the local cohort (**E**). (**F**–**J**) The confusion matrices of the LR model in the training cohort (**F**), GSE45885 cohort (**G**), GSE45887 cohort (**H**), GSE145467 cohort (**I**), and the local cohort (**J**). Abbreviation: LR: logistic regression.

First, an LR diagnosis model was constructed as follows: Score = 10.101–16.148 × *EXP*(*NCBP*2) + 0.046 × *EXP*(*DDX*20), where the EXP meant the mRNA expression value of the gene. The predictive ability of the LR model in the training cohort was quite high (AUC = 0.955, 95% CI = 0.865–1.000, Figure 6A). However, its performance in the GSE45885 cohort (AUC = 0.514, 95% CI = 0.256–0.772, Figure 6B) and the GSE45887 cohort (AUC = 0.531, 95% CI = 0.267–0.795, Figure 6C) was non-ideal. The AUCs of the LR model in the GSE145467 and local cohorts are 0.700 (95% CI = 0.493–0.907, Figure 6D) and 0.597 (95% CI = 0.465–0.729, Figure 6E), respectively. The confusion matrices of the LR model in these cohorts are shown in Figure 6F–6J, respectively. Generally, the predictive ability of the LR model was far from satisfactory, especially in the external validation cohorts, enlightening us to utilize more tools to construct the diagnosis models.

**Figure 7:**
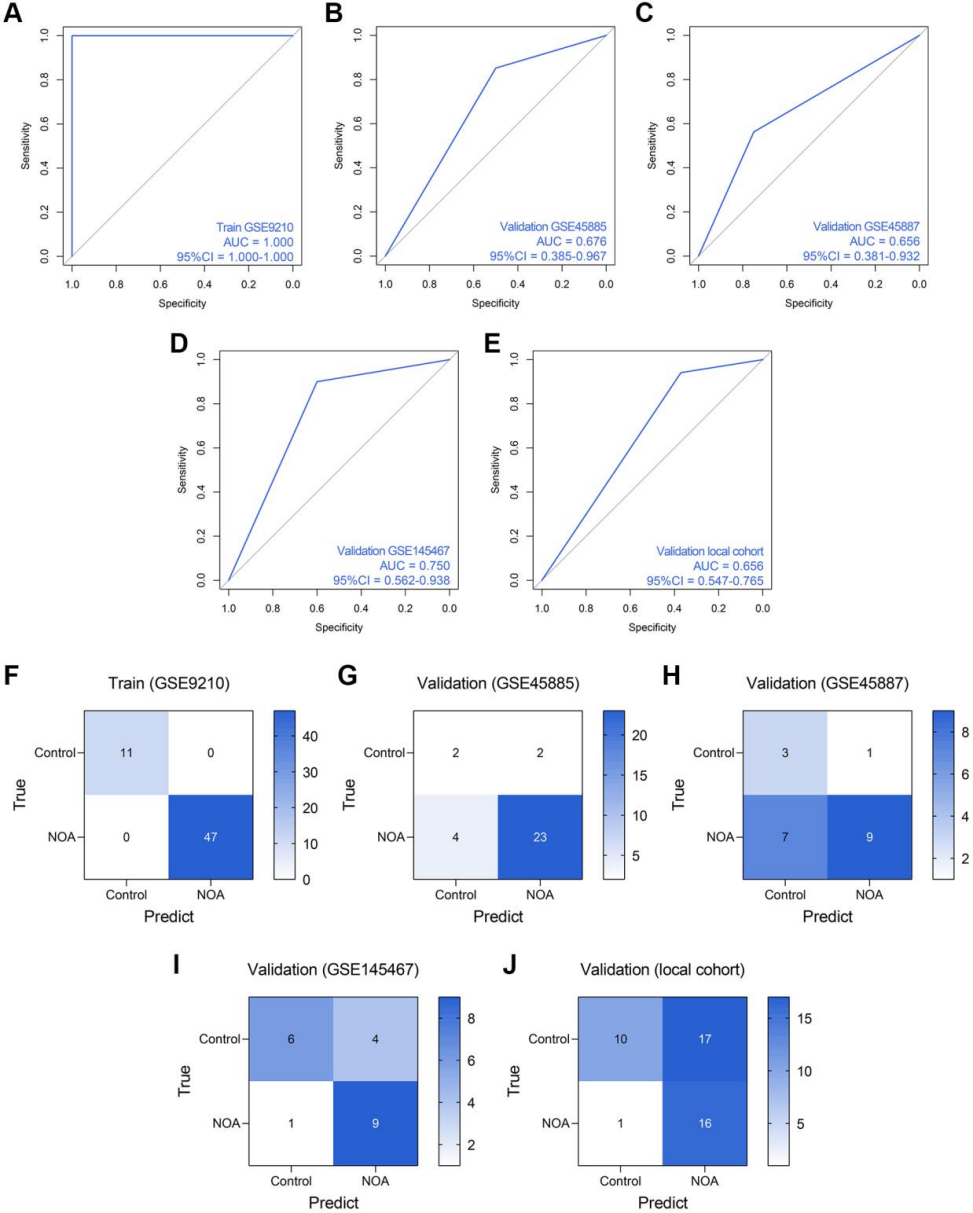
**The predictive performance of an RF diagnosis model in each cohort.** (**A**–**E**) The ROC analyses of the RF model in the training cohort (**A**), GSE45885 cohort (**B**), GSE45887 cohort (**C**), GSE145467 cohort (**D**), and the local cohort (**E**). (**F**–**J**) The confusion matrices of the RF model in the training cohort (**F**), GSE45885 cohort (**G**), GSE45887 cohort (**H**), GSE145467 cohort (**I**), and the local cohort (**J**). Abbreviation: RF: random forest.

Subsequently, we established an RF model to classify the NOA samples. The RF model showed superiority to the routine LR model in the training dataset (AUC = 1.000, 95% CI = 1.000–1.000, Figure 7A), GSE45885 dataset (AUC = 0.676, 95% CI = 0.385–0.967, Figure 7B), GSE45887 dataset (AUC = 0.656, 0.381–0.932, Figure 7C), GSE145467 dataset (AUC = 0.750, 95% CI = 0.562–0.938, Figure 7D), and local cohort (AUC = 0.656, 95% CI = 0.547–0.765, Figure 7E). Figure 7F–7J represent the confusion matrices of the RF model in each cohort.

**Figure 8:**
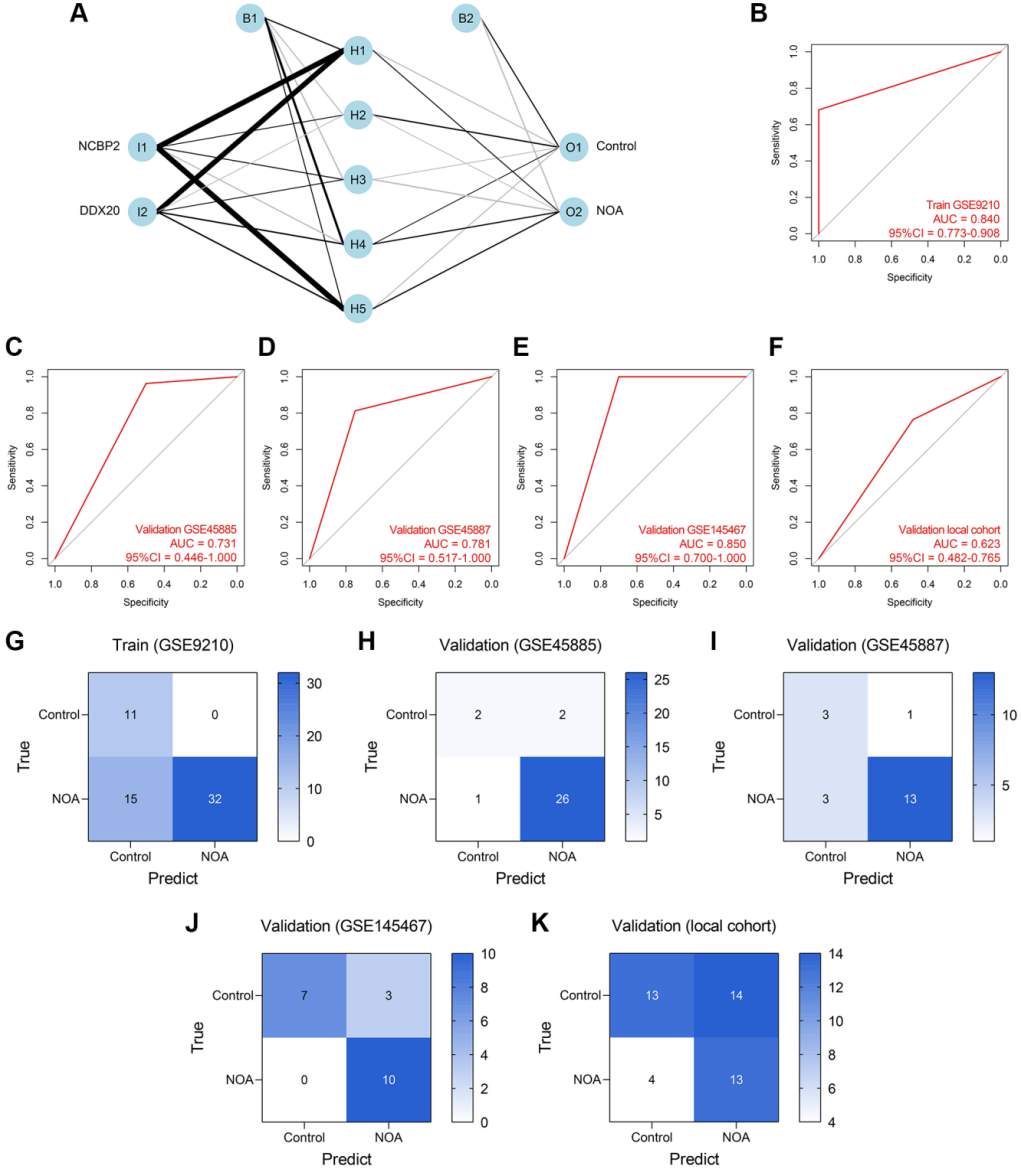
**Establishment and validation of an ANN diagnosis model.** (**A**) An ANN model containing one input layer, one hidden layer, and one output layer was constructed to diagnose NOA. (**B**–**F**) The ROC analyses of the ANN model in the training cohort (**B**), GSE45885 cohort (**C**), GSE45887 cohort (**D**), GSE145467 cohort (**E**), and the local cohort (**F**). (**G**–**K**) The confusion matrices of the RF model in the training cohort (**G**), GSE45885 cohort (**H**), GSE45887 cohort (**I**), GSE145467 cohort (**J**), and the local cohort (**K**). Abbreviation: ANN: artificial neural network.

ANN was also a widely-used method for diagnosis model establishment, and many ANN diagnosis models have been proposed and exhibited high reliability and precision in multiple diseases [20–22]. Thus, we then developed an ANN diagnosis model based on the expressions of DDX20 and NCBP2 in NOA, as displayed in Figure 8A. Similar to those previous contributions, the established ANN model showed high predictive performance across the training cohort (AUC = 0.840, 95% CI = 0.773–0.908, Figure 8B), GSE45885 cohort (AUC = 0.731, 95% CI = 0.446–1.000, Figure 8C), GSE45887 cohort (AUC = 0.781, 95% CI = 0.517–1.000, Figure 8D), GSE145467 cohort (AUC = 0.850, 95% CI = 0.700–1.000, Figure 8E), and local cohort (AUC = 0.623, 95% CI = 0.482–0.765, Figure 8F). Figure 8G–8K displayed the confusion matrices of these cohorts. The performance of the ANN model in the local cohort was not satisfying (AUC < 0.7), but we held that the result was still acceptable considering the different sample types and gene expression detection methods. As a whole, the ANN model was a promising tool to classify the NOA samples on the background of the high heterogeneity among different cohorts.

Here, we measure the predictive performance of these models mainly from the aspect of AUC. However, the other assessment indexes, containing accuracy, precision, recall, F-measure, sensitivity, specificity, positive predictive value, and negative predictive value, were also provided for reference, as listed in Table 4.

**Table 4:** The predictive performance of the established models in each cohort.

**Cohort**	**Accuracy**	**Precision**	**Recall**	**F-measure**	**Sensitivity**	**Specificity**	**Positive predictive value**	**Negative predictive value**
Logistic regression model								
GSE9210	0.983	0.979	1.000	0.989	1.000	0.909	0.979	1.000
GSE45885	0.710	0.875	0.778	0.824	0.778	0.250	0.875	0.143
GSE45887	0.700	0.813	0.813	0.813	0.813	0.250	0.813	0.250
GSE145467	0.700	0.667	0.800	0.727	0.800	0.600	0.667	0.750
Local Cohort	0.545	0.452	0.824	0.583	0.824	0.370	0.452	0.769
Random forest model								
GSE9210	1.000	1.000	1.000	1.000	1.000	1.000	1.000	1.000
GSE45885	0.806	0.920	0.852	0.885	0.852	0.500	0.920	0.333
GSE45887	0.600	0.900	0.563	0.692	0.563	0.750	0.900	0.300
GSE145467	0.750	0.692	0.900	0.783	0.900	0.600	0.692	0.857
Local Cohort	0.591	0.485	0.941	0.640	0.941	0.370	0.485	0.909
Artificial neural network model								
GSE9210	0.741	1.000	0.681	0.810	0.681	1.000	1.000	0.423
GSE45885	0.903	0.929	0.963	0.945	0.963	0.500	0.929	0.667
GSE45887	0.800	0.929	0.813	0.867	0.813	0.750	0.929	0.500
GSE145467	0.850	0.769	1.000	0.870	1.000	0.700	0.769	1.000
Local Cohort	0.591	0.481	0.765	0.591	0.765	0.481	0.481	0.765

### The functions of the DDX20- and NCBP2-asociated genes

**Figure 9:**
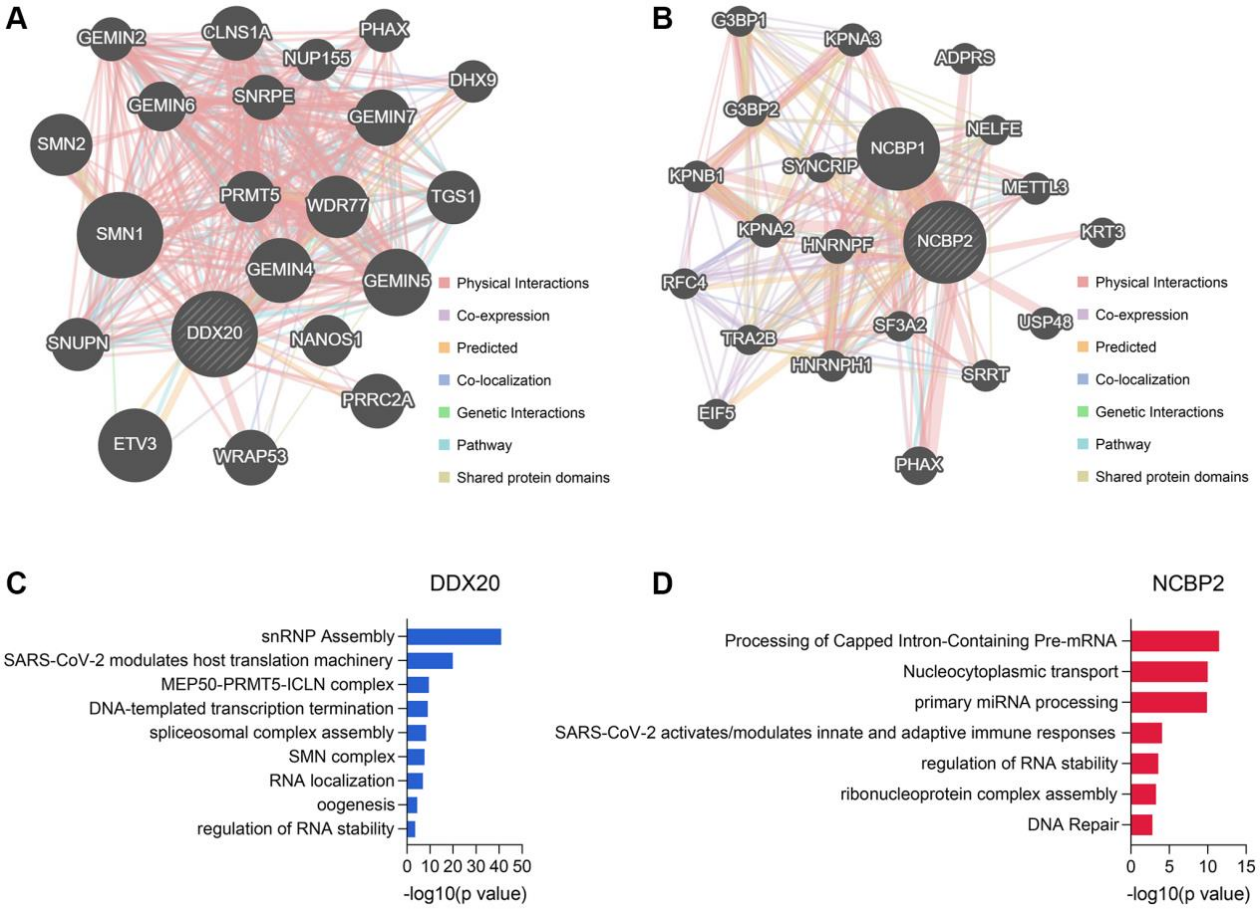
**The genes associated with DDX20 and NCBP2 and their functional enrichment.** (**A**, **B**) The Top 20 genes showing the closest connection with DDX20 (**A**) and NCBP2 (**B**). (**C**, **D**) The functional annotation of the DDX20- (**C**) and NCBP2-associated (**D**) genes, which was obtained from the Metascape database.

The Top 20 genes showing the highest connection with DDX20 and NCBP2 are displayed in Figure 9A and 9B, respectively, along with their interaction patterns. The DDX20-associated genes mainly involved cellular transcription, RNA modification, RNA splicing, RNA localization, and RNA stability maintenance (Figure 9C). The NCBP2-associated genes were mainly enriched in mRNA and miRNA processing, RNA stability regulation, and DNA repair (Figure 9D). These data revealed clues further to elucidate the biological functions of DDX20 and NCBP2.

## DISCUSSION

The rapid development of gene sequencing technologies and the tremendous advancement of computational biology and machine learning algorithms help to improve our understanding of the genetic biomarkers, associated pathogenesis, and latent therapeutic targets in multiple reproductive diseases covering a broad spectrum of prostate cancer [23], spontaneous miscarriage [24], testicular cancer [25], and NOA [26]. Investigating novel biomarkers from a particular aspect, such as transcriptional factor [27] and macrophage polarization [28], has become a popular and effective maneuver. However, the studies about the expression profiles and predictive values of RBPs are rarely seen in NOA for the moment despite the nonnegligible effects of RBPs on spermatogenesis, as discussed above. Hence, seeking more genetic biomarkers based on RBP-related genes is urgently demanded on the background of our poor knowledge of the mechanisms of NOA.

Herein, we utilized the 58 testicular samples inclusive of 11 control and 47 NOA cases from the GEO as the training cohort. 51 of 1542 RBPs reported by previous studies were differentially expressed between the control and NOA subjects. DDX20 and NCBP2 were ultimately determined as the significant features through the PPI network analysis, LASSO regression, SVM-RFE, and Boruta. Subsequently, we collected the clinical samples from 27 control and 17 NOA patients in the local hospital to verify the expression divergence of DDX20 and NCBP2. Intriguingly, we found that DDX20 and NCBP2 were significantly down-regulated in the seminal plasma samples, in addition to the testicular samples, of NOA subjects, suggesting that DDX20 and NCBP2 could serve as potential non-invasive diagnostic biomarkers. The scRNA-seq analysis of 432 testicular cell samples isolated from NOA patients indicated that DDX20 and NCBP2 were both associated with the spermatogenetic process, re-confirming the pivotal roles DDX20 and NCBP2 played in NOA.

DDX20 encoded a DEAD box protein and was first reported as an RBP interacting with miR-140-3p by Takata and his colleagues [29]. DEAD box proteins, represented by VASA, a widely-accepted germ-line specific marker, were considered critical regulatory factors in spermatogenesis via modulating multiple RNA metabolism processes [30]. The other DEAD box proteins involved in spermatogenesis include DDX3 [31], DDX25 [32], DDX23 [33], and MEL-46 [34]. Here, we first found that the disturbance of DDX20 was correlated with the spermatogenetic process, and its expressions in the seminal plasma and testes could act as a diagnostic biomarker in NOA, broadening our knowledge of the DEAD box protein family in spermatogenesis. The protein encoded by NCBP2 has an RNP domain commonly found in RBPs and was regarded as a regulator in DNA damage and repair, cell cycle, and cellular apoptosis [35]. However, the association between NCBP2 and spermatogenesis remains unclear. In all, we first found that DDX20 and NCBP2 could serve as biomarkers in NOA, shedding novel insights into the pathogenesis from an angle of RBP.

The reduced cost of gene sequencing renders the genetic diagnosis of NOA to attract increasing attention, and many great efforts have been paid. For example, Kherraf et al. employed whole-exome sequencing to construct a 7-gene panel to improve the classification of NOA, helping the patients receive a clearer diagnosis [36]. Given the satisfying performance of machine learning algorithms, especially ANN, in various diseases [37, 38], we then used LR, RF, and ANN to construct the diagnosis models based on the mRNA expression levels of DDX20 and NCBP2 and validated and compared their predictive ability in the training cohort, 3 external public validation datasets, and the local cohort. Similar to those previous works, the ANN model was observed to exhibit the highest predictive ability on average. It is worth mentioning that to the best of our knowledge, no ANN model based on genetic biomarkers has been constructed in NOA up to now. Our study reveals that ANN modelling has great potency in NOA diagnosis and deserves more attention.

The flaws of the present study should also be acknowledged. First, only the protein levels of DDX20 were detected in the clinical samples, and the detection of the protein levels of NCBP2 was lacking due to the limited financial support. Second, although the models established in this study have been verified in 4 public datasets and clinical samples, a prospective, multi-center, and large-scale clinical trial would be more beneficial to clarify the usefulness of the models. Third, our study analyzed the expression profiles, diagnosis values, and spermatogenetic association of DDX20 and NCBP2 in NOA, but their concrete biological functions in spermatogenesis remain unclear. A deeper experimental exploration is required to better elucidate the associated mechanisms in the near future.

Collectively, an ANN diagnosis model to NOA based on RBP DDX20 and NCBP2 was presented, which was externally validated in multiple public datasets and clinical samples, providing the possible cut-in points to clarify the pathogenesis and a promising tool in clinical practice.

## Supplementary Materials

Supplementary Table 1

Supplementary Tables 2-4
