# Supplementary material for: An artificial neural network model to diagnose non-obstructive azoospermia based on RNA-binding protein-related genes
Source: Aging (Albany NY). 2023 Apr 24;15(8):3120–40. doi: 10.18632/aging.204674 (PMC10188335; doi:10.18632/aging.204674)
Supplement: Supplementary Tables 2-4 [file aging-15-204674-s002.pdf]

SUPPLEMENTARY TABLES

Supplementary Table 2. 51 RBPs showing the RNA expression difference between control and NOA samples.

RBPs	logFC	AveExpr	*t*	*P*-value	FDR	B
NCBP2	–1.164687726	–1.01510361	–15.65914545	1.83E-22	1.90E-19	40.84652815
DDX20	–1.981351445	–1.256276019	–15.4569828	3.36E-22	1.90E-19	40.24705778
PSMA6	–1.04580801	–0.593954932	–14.11702322	2.16E-20	7.40E-18	36.14151813
CCDC86	–1.052820552	–1.579661768	–14.05676552	2.62E-20	7.40E-18	35.95141099
TSN	–1.556463958	–1.458954503	–13.4536697	1.84E-19	4.17E-17	34.02233281
GEMIN4	–1.089842819	–1.39742396	–12.61506455	3.01E-18	4.25E-16	31.25990374
CPSF3	–1.053481046	–0.269150663	–12.55089869	3.74E-18	4.70E-16	31.0447018
EIF5A2	–2.465618315	–2.041042187	–12.45715696	5.14E-18	5.81E-16	30.7293308
DZIP1	–1.672745206	–0.779102049	–12.40795237	6.07E-18	6.25E-16	30.56333073
TDRD7	–1.368686437	–1.133101657	–12.09505473	1.78E-17	1.44E-15	29.50028074
RPL39L	–1.521431228	–1.676756631	–11.89177303	3.59E-17	2.14E-15	28.80279562
ZNF473	–1.54377798	–1.342913734	–11.82184714	4.58E-17	2.47E-15	28.56163306
SAMD4A	–1.80946998	–1.834060238	–11.79041026	5.11E-17	2.63E-15	28.45300701
KHDRBS3	–1.368138893	–1.349001538	–11.73163021	6.28E-17	3.09E-15	28.24955889
SRPK2	–2.073289186	–1.444866371	–11.52317684	1.30E-16	5.90E-15	27.52449873
HABP4	–1.087948044	–1.004264542	–11.31664897	2.71E-16	1.06E-14	26.80069981
CARHSP1	–1.246263907	–1.237840966	–11.26739822	3.22E-16	1.21E-14	26.62730444
HINT3	–1.401923087	–1.583747782	–11.13008743	5.26E-16	1.86E-14	26.14228678
YBX2	–1.576988412	–1.725600589	–10.92210224	1.11E-15	3.48E-14	25.40321439
LARP6	1.079538654	0.630573824	10.86291927	1.37E-15	4.08E-14	25.19194891
MRPL42	–1.339480172	–1.005859006	–10.53158899	4.55E-15	1.12E-13	24.00151447
LSM14B	–2.290982508	–2.204647215	–10.15495172	1.81E-14	3.58E-13	22.63298213
EXOSC9	–1.288382785	–0.439625159	–10.06058765	2.56E-14	4.74E-13	22.28765618
FXR1	–1.018466865	–0.390312658	–10.02650068	2.90E-14	5.29E-13	22.16268071
CALR3	–2.133446923	–1.657728028	–9.922753033	4.26E-14	7.30E-13	21.78155103
TRIM56	1.035837998	0.947452277	9.08252601	9.94E-13	1.14E-11	18.65637084
RANBP17	–1.038119382	–0.834252701	–9.034483444	1.19E-12	1.34E-11	18.4758085
RNF17	–1.096010711	–0.419352204	–8.950511996	1.64E-12	1.72E-11	18.15978426
NUDT21	–1.320680742	–1.348681463	–8.947807388	1.66E-12	1.72E-11	18.1495967
FAM46A	1.105819374	1.036928608	8.783675358	3.09E-12	2.98E-11	17.5303634
MRPS15	–1.154592926	–1.112376746	–8.571113319	6.96E-12	6.05E-11	16.72572174
EZH2	–1.625135047	–1.422316674	–8.530601646	8.13E-12	6.76E-11	16.57205215
PTBP2	–1.177276056	–0.725024783	–8.290871874	2.04E-11	1.59E-10	15.66089028
YBX1	–1.082648888	–1.272797356	–8.23570308	2.52E-11	1.91E-10	15.45080955
ZFP36L2	1.2309675	0.937835892	8.008135343	6.04E-11	4.17E-10	14.5829598
G3BP2	–1.243105015	–1.079792805	–7.962686977	7.20E-11	4.82E-10	14.40942825
RUVBL2	–1.186127446	–1.587202473	–7.878862924	9.95E-11	6.46E-10	14.08922302
MAEL	–1.868901115	–1.771197607	–7.831033391	1.20E-10	7.65E-10	13.90644116
WDR5	–1.359031222	–1.727862331	–7.777332285	1.47E-10	9.15E-10	13.70116652

THUMPD3	–1.252777472	–0.923589423	–7.544794643	3.62E-10	2.08E-09	12.81185111
AARSD1	–1.008879832	–1.189050641	–7.384985781	6.71E-10	3.63E-09	12.20058137
MYEF2	–1.368124041	–0.701933592	–7.053598831	2.41E-09	1.17E-08	10.93417168
DRG1	–1.158392434	–1.473746051	–6.97794412	3.23E-09	1.52E-08	10.64549489
PSIP1	–1.010477262	–0.394447535	–6.918768578	4.06E-09	1.86E-08	10.41986273
PIWIL1	–1.230109401	–0.92726326	–6.874058726	4.83E-09	2.16E-08	10.24949506
DAZL	–1.973456426	–1.623255357	–6.589229654	1.45E-08	6.10E-08	9.166869481
DDX43	–2.054685325	–1.066758398	–6.086147677	9.91E-08	3.57E-07	7.271458078
DDX4	–2.37842902	–1.352155656	–4.899463425	8.11E-06	2.10E-05	2.960449039
RNASE11	–1.722923321	–1.272160398	–4.208908711	9.06E-05	0.000197766	0.625916397
RDM1	–1.173722882	–1.147959994	–4.196040207	9.46E-05	0.000205521	0.584080555
DDX25	–1.434031937	–1.312842028	–3.823569584	0.000324367	0.000647018	–0.595832854

Supplementary Table 3. The importance of the genes in the PPI network.

RBPs	MCC	DMNC	MNC	Degree	EPC	Bottle Neck	Ec Centricity	Closeness	Radiality	Betweenness	Stress	Clustering Coefficient
PIWIL1	252	0.5854	7	12	15.044	16	0.18919	20.28333	5.67568	295.34444	646	0.28788
DAZL	247	0.5854	7	8	14.183	2	0.18919	17.23333	5.31399	85.76111	176	0.57143
DDX4	128	0.4756	7	7	14.187	3	0.18919	17.15	5.36963	53.49444	152	0.61905
RNF17	241	0.6657	6	7	14.002	2	0.15766	16.38333	5.14706	43.96667	92	0.66667
YBX1	8	0.2842	4	6	12.307	6	0.23649	16.75	5.48092	146.27222	260	0.26667
FXR1	10	0.2378	6	6	13.011	6	0.23649	17.5	5.62003	93.7	234	0.33333
TDRD7	240	0.6657	6	6	13.822	1	0.15766	15.63333	5.06359	1.06667	8	0.93333
MAEL	240	0.6657	6	6	13.78	1	0.15766	15.63333	5.06359	1.06667	8	0.93333
YBX2	10	0.3789	4	6	12.914	8	0.18919	16.86667	5.42528	162.26667	428	0.26667
NCBP2	8	0.309	3	6	10.874	4	0.18919	15.4	5.11924	138.90556	286	0.26667
LSM14B	7	0.309	3	6	12.595	4	0.18919	16.48333	5.34181	152.85556	382	0.2
RUVBL2	5	0	1	5	8.256	8	0.18919	15.78333	5.31399	254.75	366	0
MRPS15	5	0.3078	2	5	7.4	6	0.18919	15.11667	5.14706	262.91667	474	0.1
DDX20	8	0.2593	5	5	12.672	1	0.18919	16.65	5.42528	55.15556	160	0.4
DDX25	120	0.6483	5	5	13.306	1	0.15766	14.83333	4.95231	0	0	1
HABP4	4	0.3078	2	4	11.075	9	0.23649	15.91667	5.4531	208.41111	442	0.16667
WDR5	4	0.3078	2	4	10.185	9	0.18919	16.11667	5.4531	159.86111	260	0.16667
GEMIN4	6	0.2842	4	4	11.468	3	0.18919	15.9	5.34181	33.04444	94	0.5
PSIP1	3	0	1	3	8.829	4	0.18919	14.15	5.09141	56.17778	114	0
G3BP2	3	0.3078	2	3	9.416	1	0.23649	14.66667	5.28617	68.66667	106	0.33333
EIF5A2	3	0.3078	2	3	4.23	2	0.15766	11.55	4.3124	66	116	0.33333
NUDT21	3	0.3078	2	3	7.723	2	0.15766	12.86667	4.70191	25.31667	54	0.33333
CPSF3	4	0.309	3	3	6.957	1	0.15766	11.76667	4.34022	3	8	0.66667
EZH2	2	0.3078	2	2	8.015	1	0.18919	13.81667	5.09141	0	0	1
RPL39L	2	0.3078	2	2	4.006	1	0.15766	11.05	4.28458	0	0	1
PSMA6	2	0	1	2	3.843	2	0.15766	11.41667	4.45151	66	84	0
ZNF473	2	0.3078	2	2	5.922	1	0.15766	10.93333	4.22893	0	0	1

CARHSP1	2	0	1	2	5.772	2	0.15766	11.66667	4.47933	66	148	0
TSN	1	0	1	1	5.426	1	0.15766	11.2	4.50715	0	0	0
RDM1	1	0	1	1	3.667	1	0.15766	10.75	4.39587	0	0	0
MRPL42	1	0	1	1	3.718	1	0.15766	10.38333	4.22893	0	0	0
PTBP2	1	0	1	1	1.357	1	0.05405	1	0.16216	0	0	0
KHDRBS3	1	0	1	1	1.357	1	0.05405	1	0.16216	0	0	0
RANBP17	1	0	1	1	2.34	1	0.13514	8.59286	3.39428	0	0	0
DRG1	1	0	1	1	2.128	1	0.13514	8.65952	3.53339	0	0	0
DZIP1	1	0	1	1	5.838	1	0.15766	11.18333	4.39587	0	0	0
EXOSC9	1	0	1	1	2.569	1	0.13514	8.78571	3.56121	0	0	0

Supplementary Table 4. The genes identified by feature selection algorithms.

Algorithm	Genes
LASSO	NCBP2, DDX20, TSN, SRPK2, CARHSP1
SVM-RFE	NCBP2, DDX20, CCDC86, TSN, CARHSP1, TDRD7
Boruta	NCBP2, DDX20, PSMA6, CCDC86, TSN, GEMIN4, CPSF3, EIF5A2, DZIP1, TDRD7, RPL39L, ZNF473, SAMD4A, KHDRBS3, SRPK2, HABP4, CARHSP1, HINT3, YBX2, LARP6, LSM14B, EXOSC9, CALR3, TRIM56, RANBP17, NUDT21, FAM46A, G3BP2, MYEF2, DDX4
